# The progression of leaf senescence is gated by the cytosolic arginine pool

**DOI:** 10.1038/s41477-026-02328-2

**Published:** 2026-06-30

**Authors:** Shah Hussain, Clément Boussardon, Olivier Keech

**Affiliations:** Department of Plant Physiology, UPSC, Umeå University, Umeå, Sweden

**Keywords:** Abiotic, Cell fate

## Abstract

Leaf senescence aims to degrade cellular components to recover valuable nutrients and reallocate them to other organs^1^. Once this remobilization is complete, cells undergo a vacuolar-type of programmed cell death^2^, ultimately leading to the death of the entire organ. But how do cells from a senescing leaf ‘know’ when to die? If the cell death process per se is initiated too early, remobilization may not be completed, rendering it futile. This suggests the presence of a ‘sensing’ mechanism that coordinates the remobilization phase with the onset of cell death during leaf senescence. Here, using *Arabidopsis thaliana* functional stay-green mutants, we show that senescing cells are wired to metabolically dissipate the cytosolic arginine pool, which otherwise represses the progression of leaf senescence. We propose a model in which a senescing cell uses this pool as a proxy for the completion of nitrogen remobilization and to accurately time the subsequent induction of cell death.

## Abstract (web summary)

This work provides compelling evidence that the progression of leaf senescence is gated by the cytosolic arginine pool and that senescing cells are genetically wired to dissipate this pool. It is proposed that the cytosolic arginine pool serves as a proxy for nitrogen remobilization efficiency, thereby enabling precise timing of the remobilization and termination phases.

## Main

Leaf senescence occurs in all terrestrial plants. The process can be triggered either by a developmental programme, for example in perennials during autumn in temperate climates, or by severe stress, such as water, light or nutrient deficiency, or pathogen attack^1^. In the current context of food security, better control of leaf senescence is a promising strategy to reduce yield losses by creating cultivars that are less sensitive to stress events, such as those expected to occur more frequently as a result of climate change^3,4^.

**Fig. 1:**
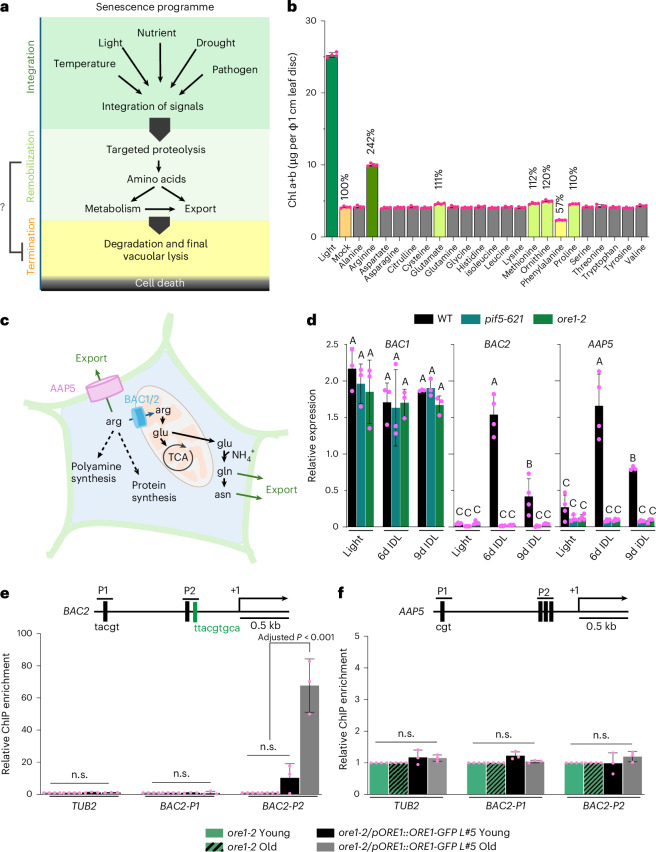
The cytosolic arginine pool is modulated by transporters induced in a senescence-dependent manner. **a**, Schematic view of the leaf senescence programme, including the question addressed in this study. **b**, Chlorophyll content in darkened leaf discs treated with amino acid (AA). Leaf discs were incubated in a buffered solution containing 1 mM of the indicated AA and kept in the dark for 6 days. Chlorophyll content was quantified, and the results are presented as mean ± s.d., *n* = 4 independent biological replicates, with individual data points as overlays. **c**, Schematic model depicting the transport of arginine (arg) to the apoplast via AAP5 or to mitochondria via BAC1 or BAC2, along with its associated metabolic pathways. **d**, Transcriptional levels of *BAC1*, *BAC2* and *AAP5* in a control leaf (Light) and in a leaf individually darkened (IDL) for 6 or 9 days. Data were obtained by qRT–PCR (primers found in Supplementary Table 1); the results are presented as mean ± s.d., *n* = 3 (*BAC1*), *n* = 4 (*BAC2* and *AAP5*) independent replicates (individual data points as overlays). Different letters indicate significant differences between genotypes by one-way analysis of variance (ANOVA) followed by a Tukey’s multiple comparisons test (*P* < 0.05). **e**,**f**, ChIP assays. ORE1 directly binds the promoter of *BAC2* but not of *AAP5*. Schematic diagrams show the 1.5-kb upstream sequences of the *BAC2* (**e**) and *AAP5* (**f**) promoters, with a translational start sites (ATG) indicated at position +1. The putative and preferred ORE1 binding sites in the P1 and P2 regions of the *BAC2* (**e**, top) and *AAP5* (**f**, top) promoters. ChIP analyses were conducted using anti-GFP antibodies with chromatin extracted from young (growth stage 1.12) and old (growth stage 8.00)^22^ *ore1-2* and *ore1-2*/*pORE1*::*ORE1-GFP#5* plants (see Supplementary Fig. 1d,e for an independent line). Subsequently, qPCR analysis was performed using ChIP DNA as a template and primers (Supplementary Table 1) specifically targeting the promoter regions of *BAC2* (**e**, bottom) and *AAP5* (**f**, bottom) genes. *TUBULIN2* (*TUB2*) was used as a control. Data are represented as mean ± s.d., *n* = 4 independent biological replicates for *ore1-2* (young/old), *n* = 3 for *ore1-2*/*pORE1*::*ORE1-GFP#5* (young/old), and individual data points as overlays. Statistical differences were assessed using two-way ANOVA followed by Tukey’s multiple comparisons test (*P* < 0.05). Source data

In essence, leaf senescence aims to degrade proteins, nucleic acids, lipids and certain cell structures to recover nutrients, particularly nitrogen, and remobilize them towards storage or newly growing organs^5^. Yet, the biological process itself is complex and requires many regulatory circuits, allowing multilevel control (for detailed reviews, see refs. ^6–8^). The progression of leaf senescence can be viewed as a three-step programme (Fig. 1a). First, there is an integration phase in which both internal and external signals are integrated into a complex transcriptional module. This integration is notably based on several signalling cascades and epigenetic events as well as multiple transcriptional and post-transcriptional regulations orchestrated by hormonal interplay^8^. Second, there is a remobilization phase during which the transcriptional feedforward loops will lead to the activation of catabolic enzymes such as proteases, lipases and nucleases, as well as specific transporters^9,10^ (Fig. 1a). In turn, these molecular actors orchestrate the targeted degradation of numerous biomolecules and contribute to the remobilization of nutrients. In particular, the proteolysis of chloroplast proteins leads to a major release of nitrogen in the form of free amino acids, which are either metabolized within the cell or exported as a nitrogen source^11,12^. Glutamine and asparagine seem to be the preferential amino acids exported and found in the phloem sap, probably due to their high N/C ratio^9^, supported by a strongly increased abundance of the transcripts coding for glutamine synthetase 1 (GS1) and asparagine synthetase 1 (ASN1) during leaf senescence^13^. Finally, when remobilization is complete, a termination phase ensues, during which several lytic enzymes accumulate in vacuoles until a final burst of these vacuoles disrupts the cell’s integrity, thus leading to its death^2^.

Although many molecular mechanisms have been deciphered for these three phases, the timing between one phase and another remains unclear. While the integration and remobilization phases are strongly intertwined, it would appear essential that the termination phase be engaged only once the remobilization phase is complete. So, how does a senescing cell know that remobilization is complete, and the termination phase should be induced? This question challenges our current understanding of the concepts of ‘timing’ and ‘sensing’ in cells undergoing the process of senescence (Fig. 1a).

We hypothesized that certain metabolites could act as metabolic sensors, thereby providing the cell with information about the progression of catabolism and the export of nutrients. Liebsch et al.^10^ indicated that knockouts of *PIF5* (*Phytochrome Interacting Factor 5*) and *ORE1* (*Oresara 1*), two transcription factors whose suppression leads to a functional stay-green (FSG) phenotype, that is, leaves that do not undergo stress-induced senescence as their wild-type (WT) counterpart, exhibited a massive accumulation of amino acids. This resulted from the combined effects of mild, targeted protein degradation and the non-induction of several amino transporters^10^. In line with these observations, we first tested whether some of these amino acids could have a repressing effect on the progression of leaf senescence. To this end, we incubated *Arabidopsis* leaf discs in a buffered aqueous solution supplemented with 1 mM of a given amino acid. The leaf discs were placed in darkness for 6 days at 22 °C (Supplementary Fig. 1a). Subsequently, the chlorophyll content of leaf discs was quantified as a proxy for the progression of senescence. Surprisingly, despite being key metabolites for nitrogen export, neither asparagine nor glutamine had a repressing effect on the progression of senescence in the leaf discs. However, leaf discs treated with arginine retained more than twice as much chlorophyll as the mock-treated discs and those treated with other amino acids. Of note, glutamate, ornithine, and proline—three amino acids located in the close metabolic vicinity of arginine (Supplementary Fig. 1b)—also had a milder yet significant effect (10–20%) on chlorophyll retention compared with mock-treated leaf discs (Fig. 1b).

In cellulo, arginine is synthesized in plastids and then exported to the cytosol, where it can follow three distinct routes^14^ (Fig. 1c):

1. Theoretically, arginine can be exported out of the cell, although this has not been extensively studied. Two transporters, AAP3 and AAP5 (AMINO ACID PERMEASE), were shown to efficiently transport arginine, as well as other amino acids, but with minor efficiency^15,16^. Yet, AAP3 is mostly expressed in roots, while AAP5 is expressed in all tissues.
2. Arginine can also be catabolized to urea or glutamate in mitochondria (Supplementary Fig. 1b). To this end, arginine is imported via BASIC AMINO ACID CARRIER 1 and 2 (BAC1 and BAC2)^17,18^.
3. Arginine can remain in the cytosol and serve as a substrate for the biosynthesis of polyamines, particularly putrescine and spermidine, or for protein synthesis (Fig. 1c and Supplementary Fig. 1b).

Mutants that will directly or indirectly affect the expression of *ORE1*, that is *ore1-2* and *pif5-621*, respectively, were shown to accumulate arginine in individually darkened leaves (IDL)^10^, a commonly used experimental set-up to induce and synchronize rapid leaf senescence^19,20^. This suggests that the capacity to transport arginine to the apoplast via AAP5 or to mitochondria via BAC1 or BAC2 could be limited in these genotypes. Looking at the transcript abundance of these transporters in IDL with quantitative reverse-transcription polymerase chain reaction (qRT–PCR) analysis revealed that there was no induction of *BAC1* in WT, *pif5-621* and *ore1-2* leaves darkened for either 6 or 9 days. However, a strong induction of *BAC2* and *AAP5* expression was noticed in WT undergoing senescence but not in the two FSG lines, supporting a senescence-dependent induction of these two arginine transporters (Fig. 1d).

Then, because the induction of arginine transporters appears to be senescence dependent, we questioned whether the *BAC2* and *AAP5* promoters could be direct targets of ORE1, a master regulator of senescence. Matallana-Ramirez et al.^21^ established a conserved motif for the ORE1 binding site: T[TAG][GA]CGT[GA][TCA][TAG]. In silico promoter analysis shows three potential ORE1 binding sites in the *BAC2* promoter, while the promoter of *AAP5* contains four CGT regions (Supplementary Fig. 1c–e). To test whether *BAC2* and *AAP5* could be direct targets of ORE1, we conducted chromatin immunoprecipitation (ChIP)–quantitative PCR (qPCR) analysis using *ore1-2* and *ore1-2*/*pORE1::ORE1-GFP* plants with anti-GFP antibody. This showed that ORE1 directly binds the *BAC2* promoter in its P3 region (Fig. 1e and Supplementary Fig. 1d). By contrast, no association of ORE1 in the *AAP5* promoter was indicated (Fig. 1f and Supplementary Fig. 1e), suggesting an indirect regulation.

**Fig. 2:**
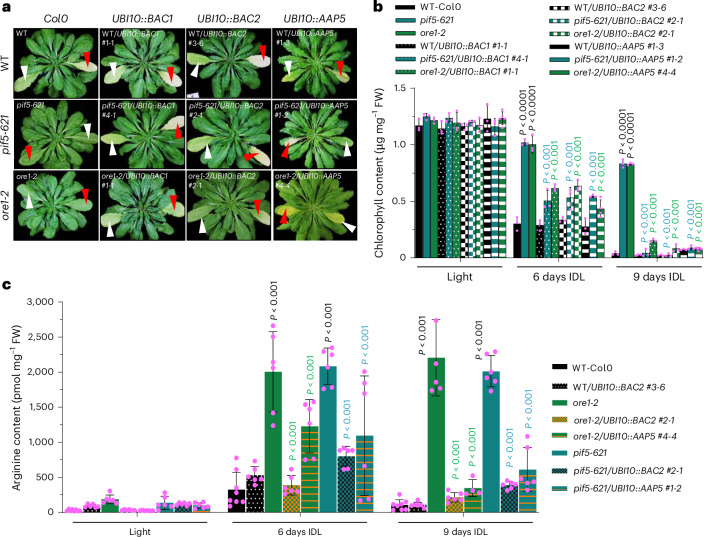
Dissipating the cytosolic arginine pool reactivates the progression of leaf senescence. **a**, Phenotype of individually-darkened leaves of WT-Col0, *pif5-621*, *ore1-2* and complemented lines expressing *UBI10*::*BAC1*, *UBI10*::*BAC2* or *UBI10*::*AAP5* in WT, *pif5-621* and *ore1-2* background. Leaves were individually darkened for 6 (white arrowhead) or 9 (red arrowhead) days. **b**, Chlorophyll in IDL of plants used in **a**. Data are represented as mean ± s.d., *n* = 3 for all lines, except *ore1-2/Ubi10::AAP5 #4-4 Light n* = 5, *ore1-2/Ubi10::AAP5 #4-4 6* *d n* = 4 and *ore1-2/Ubi10::AAP5 #4-4 9* *d n* = 5. Data represent independent biological replicates, with individual data points shown as overlays. Significant differences were determined by two-way ANOVA followed by a Tukey’s multiple comparisons test (*P* < 0.05), after comparison with WT (black), with *pif5-621* (teal) or with *ore1-2* (moss green). **c**, Arginine abundance (pmol mg^−1^ fresh weight (FW)) in WT-Col0, WT/*UBI10*::*BAC2*, *ore1-2*, *ore1-2*/*UBI10*::*BAC2*, *ore1-2*/*UBI10*::*AAP5*, *pif5-621*, *pif5-621*/*UBI10*::*BAC2* and *pif5-621*/*UBI10*::*AAP5* leaves in light and individually darkened for 6 or 9 days. Data are presented as mean values ± s.d., *n* = 6 independent biological replicates, and individual data points as overlays. Significant differences were determined by two-way ANOVA followed by a Tukey’s multiple comparisons test (*P* < 0.05), after comparison with WT (black), with *ore1-2* (moss green) or with *pif5-621* (teal). Source data

Taken together the facts that, in leaves, (1) *AAP5* and *BAC2* are induced by senescence, (2) *BAC2* is a direct target of ORE1 and (3) the addition of arginine delays senescence, we reasoned that the presence of arginine in the cytosol could act as a sensing mechanism, gating the induction of the terminal phase of leaf senescence. To test this hypothesis, we generated several independent lines constitutively expressing *AAP5*, *BAC1* or *BAC2* using a *UBI10* promoter in a *pif5-621* and *ore1-2* background. *WT/UBI10::BAC1* and *WT/UBI10::BAC2* showed a 30-fold and 17-fold increase in BAC1 and BAC2 protein abundance, respectively, as compared with the WT control (Supplementary Fig. 2a). However, the data-independent acquisition (DIA) proteomics analysis did not allow the detection of AAP5 in any of the samples, possibly due to a poor ionization efficiency or an interference with other co-eluting peptides. Nonetheless, reactivating the expression of either *AAP5* or *BAC2* reverted the FSG phenotype into a senescing phenotype for both *pif5-621* and *ore1-2* lines in IDL (Fig. 2a,b and Supplementary Fig. 2b–d), as well as during developmental leaf senescence (Supplementary Fig. 3a–c). Similarly, the constitutive expression of BAC1 also restored the progression of leaf senescence in FSG lines (Fig. 2a,b and Supplementary Figs. 2b,c and 3a–c), which indicates that: (1) the basal expression of *BAC1*, which was not significantly different between WT and FSG lines (Fig. 1d), is not sufficient to process the cytosolic arginine pool; and (2) the mitochondrial import of arginine operated by BAC transporters is essential to modulate the progression of leaf senescence. Furthermore, the efficiency of the depletion of the cytosolic arginine pool by the reintroduction of the transporters was monitored by liquid chromatography (LC)–mass spectrometry (MS). While both *pif5-621* and *ore1-2* had a massive accumulation of arginine in 6 or 9 days IDL as compared with WT, lines constitutively expressing *AAP5* or *BAC2* had a much lower abundance of arginine, confirming the functional capacity of the transporters expressed in FSG lines (Fig. 2c). It is also worth mentioning that the abundance of putrescine and spermidine did not differ between the lines after 6 days of IDL, and only a small variation in putrescine was detected in lines complemented with AAP5 after 9 days of IDL (Supplementary Fig. 3d,e).

**Fig. 3:**
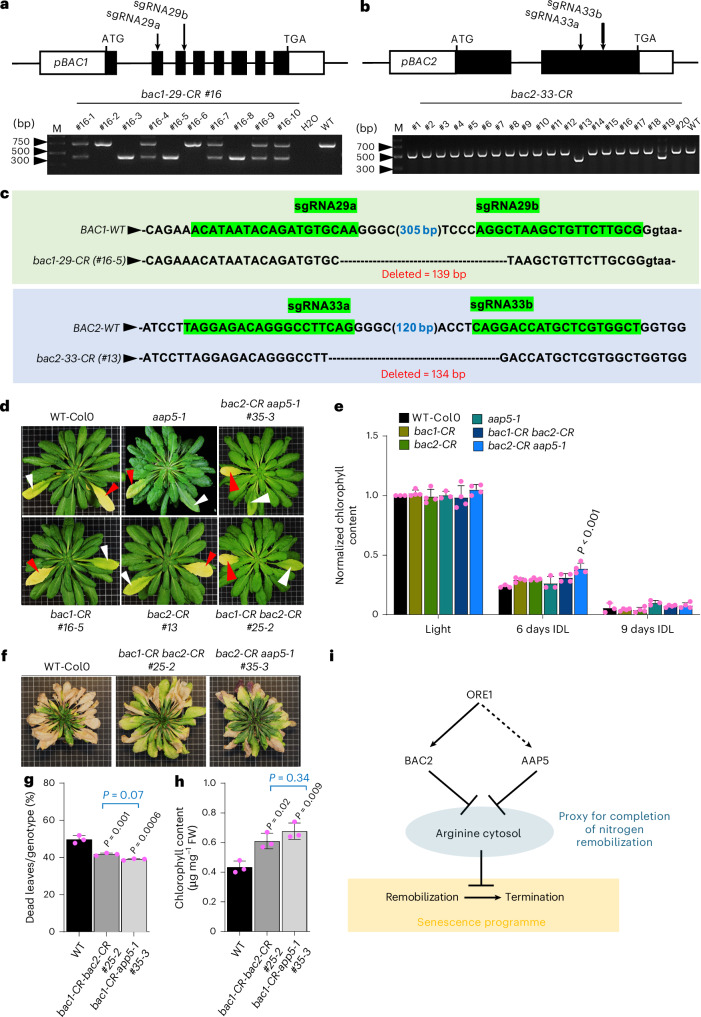
The *bac2aap5* mutant shows a delayed leaf senescence phenotype in response to darkness and developmental ageing. **a**,**b**, CRISPR–Cas9-mediated deletion in *BAC1* (**a**) and *BAC2* (**b**) genes. Schematic illustration of the double sgRNA strategy targeting the *BAC1* at exon 2 and exon 3, and *BAC2* in exon 2 (**a** and **b**, top). The promoter and terminator sequences are represented by white boxes. Genotyping PCR analysis of *bac1-29-CRISPR* lines using genomic DNA. The *bac1-29-CRISPR* lines #16-3, #16-5 and #16-8 showed a 319-bp deletion in the *BAC1* gene between two sgRNA sites, resulting in a 431-bp band (**a**). Genotyping PCR analysis of *bac2-33-CRISPR* lines was performed using genomic DNA. *bac2-33-CRISPR* line #13 showed a 134-bp deletion in the *BAC2* gene between two sgRNA sites, resulting in a 416-bp band (**b**). The designed sgRNAs for both *BAC1* and *BAC2* genes are provided in Supplementary Table 1. **c**, Confirmation of *BAC1* and *BAC2* gene editing by DNA sequencing analysis. The *bac1-29-CRISPR* line #16-5 line and *bac2-33-CRISPR* line #13 showed deletion of a 319-bp and 134-bp region between two sgRNA sites, respectively. **d**, IDL phenotype of WT-Col0, *aap5-1*, *bac1-29-CRISPR* (#16-5), *bac2-33-CRISPR* (#13) single mutants, and *bac2-CR aap5-1*(#35-3), *bac1-CR bac2-CR* (#25-2) double mutants. Leaves were individually darkened for 6 (white arrowhead) or 9 (red arrowhead) days. **e**, Chlorophyll in IDL of plants used in **d**. Data are mean normalized to WT-Col0 light samples (*n* = 3 ± s.d. for WT-Col0 and *aap5-1*; *n* = 4 ± s.d. for *bac1-CR*, *bac2-CR*, *bac1-CR* *bac2-CR* and *bac2-CR aap5-1*), independent biological replicates, and individual data points as overlays. Significant differences were determined by two-way ANOVA followed by a Dunnett’s multiple comparisons test (*P* < 0.05) relative to WT-Col0 plants. **f**, Age-related developmental leaf senescence phenotype of WT-Col0, *bac2-CR aap5-1* and *bac1-CR bac2-CR* at growth stage 8.00 (ref. ^22^) under short-day conditions. **g**, Age-dependent leaf death quantification of plants used in **f**. **h**, Chlorophyll content in the whole rosette of plants used in **f**. Data are presented as mean values; error bars indicate s.d. (*n* = 3), and individual data points are shown as overlays. Significant differences were determined by two-way ANOVA followed by a Tukey’s multiple comparisons test (*P* < 0.05), after comparison with WT (black). **i**, Working model depicting the key role of the cytosolic arginine pool in gating the transition between the remobilization and termination phases. The dynamics of this pool are genetically controlled by ORE1, which directly promotes *BAC2* transcription (solid arrow) and indirectly positively regulates *AAP5* expression (dashed arrow). Source data

Finally, we tested whether the loss of any of these arginine transporters would suffice to delay senescence in IDL and during developmental ageing. To this aim, we obtained the TDNA knockout *aap5-1* from Svennerstam et al.^15^ and concomitantly generated new clustered regularly interspaced short palindromic repeats (CRISPR)–Cas9 lines for *BAC1* and *BAC2* (Fig. 3a–c and Supplementary Fig. 4a–c). While the *bac1-CR*, *bac2-CR* and *aap5-1* single mutants did not show any delayed senescence phenotype in IDL based on chlorophyll content (Fig. 3d,e), a double mutant *bac2-CR aap5-1* showed a significantly delayed progression of senescence in IDL, but only after 6 days (Fig. 3e). However, much stronger effects were observed during developmental leaf senescence, where both *bac1-CR bac2-CR* and *bac2-CR aap5-1* mutants had a lower number of dead leaves and retained significantly higher chlorophyll content in their rosettes at developmental stage 8.00 (ref. ^22^) (Fig. 3f–h).

Collectively, these results provide compelling evidence that the cytosolic arginine pool plays a key role in modulating the progression of leaf senescence. Although the exact subcellular distribution of the arginine pool would require additional confirmations, we considered it unlikely that a contribution from the plastid pool could occur, as most genes involved in the arginine biosynthetic pathway are repressed during IDL^20^. Yet, at this stage, the presence of arginine in the vacuole could not be ruled out. Recently, arginine biosensors were developed^23,24^, and they could provide valuable insight into the respective subcellular pools; however, their efficiency in plants remains to be tested. Supporting our findings, we demonstrate that the dynamics of this pool are genetically controlled by *ORE1*, a master regulator of leaf senescence. Indeed, ORE1 directly binds to the promoter of *BAC2* and activates its transcription. In turn, an increase of BAC2 promotes the import of arginine to mitochondria where metabolic reactions lead to the production of urea, which can be exported out of mitochondria and be catabolized by urease leading to CO_2_ and NH_3_; or to reducing equivalents and glutamate, the latter providing the carbon backbone necessary for the biosynthesis of glutamine and asparagine, and hence for nitrogen export (Supplementary Fig. 1b). However, the molecular mechanism by which arginine represses the progression of leaf senescence remains to be elucidated. Interestingly, several studies in mammals have also pointed out the capacity for L-arginine to modulate metabolism and enhance the survival of T cells^25,26^. Three nuclear proteins, BAZ1, PSIP1 and TSN, which may affect DNA binding, were shown to be required for mediating L-arginine’s effect on survival^25^.

Here, we originally thought that arginine could feed the pool of polyamines, which have been shown to repress senescence^10,27^, but metabolomic data do not support this hypothesis (Supplementary Fig. 3d,e). Also, we ruled out the possibility of mitochondria playing a signalling role. Indeed, the fact that AAP5, an arginine transporter located at the plasma membrane, can also restore a senescing phenotype in FSG lines supports a key role for the cytosolic arginine rather than a modulation of the signalling by an energy-dependent mechanism. Arginine could support the production of nitric oxide (NO), a neutral and lipophilic gaseous molecule that acts as a multifunctional signalling molecule, reported to modulate plant development and stress responses^28,29^. However, the presence of nitric oxide synthase (NOS)-like enzymes in land plants remains elusive^30,31^, and additional research is required to decipher the NO-dependent signalling pathways originating from arginine. For now, we propose a model in which the senescing cells use the cytosolic arginine pool as a proxy for the completion of the remobilization of nitrogen (Fig. 3i). This would provide senescing cells with information about the efficiency of nitrogen export, thereby enabling them to appropriately time the transition between the remobilization and termination phases. In fact, the level of arginine is generally kept very low in cells under steady state^20,32^; however, an increase in the abundance of this amino acid has often been associated with a response to drought stress^33^ or even in tissue from resurrection plants subject to dehydration^34,35^. The striking possibility of reverting an FSG phenotype into a senescing phenotype also suggests that arginine could act as a metabolite that controls the point of no return, that is, the irreversibility of the process. Indeed, leaf senescence can, under certain conditions, be arrested or even reversed. This has puzzled scientists for several decades, and today controlling the point of no return appears to be a suitable strategy to minimize biomass losses. Therefore, modulating the metabolic fate of arginine in plants under stress could be an avenue for future targeted breeding to rescue trees and crops subjected to adverse conditions.

## Methods

### Plant materials and growth conditions

Seeds of *Arabidopsis thaliana* WT and mutants plants used in this study were from Columbia (Col-0) ecotype. The two FSG mutant lines, *pif5-621* and *ore1-2*, have been previously characterized by Liebsch et al.^10^, and TDNA knockout *aap5-1* was obtained from Svennerstam et al.^15^ and rechecked for the correct mutation.

Seeds from all the selected genotypes were grown under short-day conditions (8-h-light/16-h-dark photoperiod, 22 °C/17 °C), at 65% relative humidity and 150 µmol m^−2^ s^−1^ photosynthetically active radiation, or long-day conditions (16-h-light/8-h-dark photoperiod, 22 °C/17 °C), at 65% relative humidity and 180 µmol m^−2^ s^−1^ photosynthetically active radiation on a mixture of soil:vermiculite (3:1).

For IDL experiments, 7-week-old plants were used. Two leaves per plant were individually covered (IDL), as detailed in Keech et al.^19^.

### RNA extraction and qRT–PCR analysis

Total RNA was extracted from approximately 100 mg of frozen material using the Qiagen RNeasy Plant Mini Kit (Qiagen), with DNase treatment performed according to the manufacturer’s recommendations. RNA concentrations were quantified using a NanoDrop spectrophotometer. Then, 1 μg of total RNA underwent reverse transcription using the SuperScript II RNase-Reverse Transcriptase assay (Invitrogen) to prepare complementary DNA (cDNA). The qRT–PCR analysis was carried out on a CFX384 Real-Time System (Bio-Rad) using LightCycler 480 SYBR Green I Master (Roche Diagnostics). Three biological replicates were used from all selected genotypes. Primer information is presented in Supplementary Table 1.

### ChIP assay

For the ChIP assay, leaf tissues (1.0 g) from *ore1-2* and *ore1-2*/*pORE1::ORE1-GFP* (lines #5 and #6) were collected and fixed in sterilized H_2_O containing 1% formaldehyde. ChIP assays were conducted utilizing anti-GFP antibodies (Abcam 290; dilution 1:3,000) following established protocols^36,37^. qPCR analyses were carried out using CFX384 Real-Time System (Bio-Rad) and gene-specific primers (Supplementary Table 1).

### Plasmid construction and generation of transgenic lines

cDNA of *BAC1*, *BAC2* and *AAP5* was synthesized and amplified from WT/Col-0 RNA using primers containing attB recombination sites for Gateway cloning (Supplementary Table 1). PCR products were cloned into the pDONR207 donor vector by BP cloning and then inserted in pUB-DEST (UBQ10 promoter, no tag) destination vectors. Destination vectors containing *UBI10::BAC1*, *UBI10::BAC2* and *UBI10::AAP5* were used for *Agrobacterium tumefaciens* (GV3101::pMP90)-mediated transformation of WT/Col-0 and mutants plants using the floral-dipping method^38^. Transgenic plants were selected using Basta (10 μg ml^−1^). Homozygous T_3_ lines were used to perform experiments.

### Chlorophyll measurements

For dark-induced leaf senescence and amino acid feeding experiments, tissue samples were collected and immediately frozen in liquid nitrogen. About 10–25 mg of leaf powder was used. Chlorophyll was extracted in 80% acetone. Samples were then vortexed and incubated for 15 min in the dark. After incubation, samples were centrifuged at 15,000*g* for 15 min at 4 °C. The absorbance of the supernatant was measured at 750 nm to account for background, at 663 nm to quantify chlorophyll *a* and at 647 nm to quantify chlorophyll *b* using a Shimadzu 2600i spectrophotometer. Finally, the chlorophyll content was calculated using Chla+b = 7.15(absorbance (*A*)_663_ – *A*_750_) + 18.71(*A*_647_ – *A*_750_) (μg ml^−1^) (ref. ^39^).

### Amino acid feeding assay

Leaf discs (1 cm in diameter) from WT/Col-0 plants were incubated in 1 mM solutions of respective amino acids and in a mock solution (1 mM NaH_2_PO_4_ buffer, 0.025% Tween-20) as previously described by Liebsch et al.^10^.

### Construction of CRISPR plasmids and generation of *BAC1* and *BAC2* mutant lines

Generation of CRISPR lines was carried out as described by Goretti et al.^40^. In brief, single guide RNAs (sgRNAs) specifically targeting *BAC1* and *BAC2* were identified using Benchling (https://www.benchling.com/) and CRISPR-P 2.0 (http://crispr.hzau.edu.cn/CRISPR2/). A PCR-based technique was used to construct the CRISPR cassette, which contains a promoter, two sgRNAs, Cas9 coding sequence, a selection marker and a terminator. The CRISPR cassettes were then assembled to destination vector using GreenGate reaction (150 ng of each component, 2 µl FastDigest buffer, 2 µl of 10 mM ATP, 1 µl of 30 U ml^−1^ T4 ligase and 1 µl Eco31I in a 15-µl reaction) in 50 cycles of 5 min restriction/ligation at 37 °C and 16 °C, respectively, followed by 5 min at 50 °C and 5 min at 80 °C. All destination vectors were amplified in *Escherichia coli* strain *DH5a* and confirmed with sequencing (Eurofins). Destination vectors containing different combinations of sgRNAs were then transformed into WT/Col-0 plants using the floral-dipping method^38^. Stable CRISPR mutants were selected using amplification fragment length polymorphism, DNA sequencing and RT–PCR analysis. Information about sgRNAs is presented in Supplementary Table 1.

The double mutants *bac1-29-CR bac2-33-CR* and *bac2-33-CR aap5-1* were generated by crossing the respective homozygous single mutants and self-pollination of the F1 generation. Homozygous double mutants were identified using genotyping PCR; primers are listed in Supplementary Table 1.

### Proteomic analysis

#### Protein extraction, clean-up and digestion

Tissue samples were collected from 6-week-old WT-Col-0 plants expressing *UBI10::BAC1*, *UBI10::BAC2* and *UBI10::AAP5* constructs and immediately frozen in liquid nitrogen. About 25 mg of leaf powder from three biological replicates was used for protein extraction.

Leaves tissues were lysed in 200 µl of lysis buffer containing 4% sodium dodecyl sulfate, 50 mM HEPES (pH 7.6) and 1 mM dithiothreitol, then heated to 95 °C. After centrifugation, the clarified supernatants were collected for protein quantification and digestion. A total of 50 µg of protein per sample was reduced by incubation at 37 °C for 30 min in the same lysis buffer. Reduction was followed by alkylation using 10 mM chloroacetamide at room temperature for 10 min. Samples were processed using a modified SP3 cleanup and digestion protocol^41,42^. In brief, 40 µl of Sera-Mag SP3 bead mix was added to each sample along with 100% acetonitrile to reach a final concentration of 70% acetonitrile. The mixture was incubated at room temperature under rotation for 20 min. Beads were then captured on a magnetic rack, and the supernatant was discarded. Beads were washed twice with 70% ethanol and once with 100% acetonitrile. The bead-bound proteins were resuspended in 100 µl digestion buffer (50 mM HEPES, pH 7.6, 10% acetonitrile and 1 mM CaCl_2_) containing 1 µg RapiZyme Trypsin MS Grade (Waters) and digested overnight at 37 °C.

The following day, 100% acetonitrile was added to the peptide solution to a final concentration of >95%. After pipette mixing and a 20-min incubation at room temperature, peptides were separated from the beads using a magnetic rack. Beads were washed with 500 µl of acetonitrile, then resuspended in 100 µl of 0.1% formic acid in water. Following magnetic separation, the peptide-containing supernatant was transferred to low-binding tubes for LC–MS injection.

#### LC–electrospray ionization–MS/MS

For MS analysis, 500 ng peptides were loaded onto Evotip PURE (EV2013) according to the manufacturer’s instructions (EvoSep). The samples were separated using a 30SPD gradient on the Evosep system. The analytical column is equilibrated at 1,500 nl min^−1^. The gradient went from 0% B up to almost 40% B with 220 nl min^−1^ flow, followed by an increase to 1,500 nl min^−1^ for washing. The separation column was a PepSep FIFTEEN 15 cm × 150 µm × 1.5 µm (Bruker 1893474) connected to a 20 µM ZDV Sprayer (Bruker 1865710) at 40 °C. Mobile phase A consisted of 0.1% formic acid in Milli-Q water, and mobile phase B consisted of 0.1% formic acid in acetonitrile.

Online LC–MS was performed using a Tims TOF HT mass spectrometer (Bruker) using the CaptiveSpray source, with a capillary voltage of 1,500 V, dry gas flow of 3 l min^−1^ and dry gas temperature of 180 °C. Collision energy was set to 20 eV for 1/*k*_0_ = 0.60 V s cm^−2^ and 59 eV for 1/*k*_0_ = 1.60 V s cm^−2^. Data were acquired using TimsControl v 6.0.6 and Compass HyStar 6.3.1.8. The above conditions were used for DIA diaPASEF (parallel accumulation-serial fragmentation). For diaPASEF analysis, precursor ions were selected in the range of 300–1,300 *m*/*z* and 0.6–1.6 1/*k*_0_ ion mobility. Optimized isolation windows based on the Bruker human library with the assistance of the Py-DiaId Python package^43^ were implemented. Collision energies for fragmentation were ramped from 15 to 70 eV. Parallel accumulation and serial fragmentation were performed with a cycle time of 1.8 s.

Raw data files were analysed using Spectronaut (Biognosys) with the DirectDIA workflow for label-free quantification. DirectDIA enables efficient analysis by generating a project-specific spectral library directly from the DIA files, bypassing the need for a pre-existing library. Data search parameters included a 1% false discovery rate at both the protein and peptide levels, a maximum of two missed cleavages, and trypsin as the enzyme and cleavage specificity. The search was performed against the UniProt *Arabidopsis thaliana* reference proteome (UP000006548) database. Protein quantification was based on the major group top 3 and minor group top 6, and using only peptides for protein quantification that map to a single gene ID.

### Metabolite analysis

#### Standards and calibration curve

Amino acid and polyamine standards (alanine, arginine, aspartic acid, cysteine, glutamic acid, glycine, histidine, isoleucine, leucine, lysine, methionine, phenylalanine, proline, serine, threonine, tyrosine, valine, citrulline, ornithine, tryphtophan and norvaline) were obtained from Cambridge Isotope Laboratories. Glutamine, asparagine, GABA, kynurenine, 5-hydroxytryptophan, putrescine, *N*-acetyl spermidine, spermine, spermidine, reserpine and norvaline were obtained from Sigma-Aldrich. Isotopically labelled amino acid standards (alanine (^13^C_3_, ^15^N), arginine (^13^C_6_, ^15^N_4_), aspartic acid (^13^C_4_, ^15^N), cystine (^13^C_6_, ^15^N_2_), glutamic acid (^13^C_5_, ^15^N), glycine (^13^C_2_, ^15^N), histidine (^13^C_6_, ^15^N_3_), isoleucine (^13^C_6_, ^15^N), leucine (^13^C_6_, ^15^N), lysine (^13^C_6_, ^15^N_2_), methionine (^13^C_5_, ^15^N), phenylalanine (^13^C_9_, ^15^N), proline (^13^C_5_, ^15^N), serine (^13^C_3_, ^15^N), threonine (^13^C_4_, ^15^N), tyrosine (^13^C_9_, ^15^N), valine (^13^C_5_, ^15^N) and putrescine (^13^C_4_)) were obtained from Cambridge Isotope Laboratories. Citrulline (d_4_), GABA (^13^C_4_), glutamine (^13^C_5_), asparagine (^13^C_4_), ornithine (d_6_), tryptophan (d_8_) and kynurenine (d_4_) were obtained from Sigma-Aldrich. Stock solutions of each compound were prepared at a concentration of 500 ng μl^−1^ and stored at −80 °C. A 10-point calibration curve (0.01–100 pmol µl^−1^) was prepared by serial dilutions and spiked with internal standards at a final concentration of 5 pmol μl^−1^.

MS-grade formic acid was purchased from Sigma-Aldrich and high-performance LC-grade acetonitrile from Fisher Scientific.

#### Extraction of amino acids and polyamines

About 10 mg of plant powder from *Arabidopsis* leaf tissue was used for the analysis of amino acids and polyamines. Amino acids and polyamines were extracted by adding 750 µl of extraction solution (methanol:chloroform, 60:20:20, v/v/v) containing internal standard norvaline at 0.2 pmol μl^−1^. The samples were shaken with a tungsten bead in a mixer mill at 30 Hz for 3 min, the bead was removed, and the samples were centrifuged at +4 °C, 14,000 rpm (18,620*g*), for 10 min. The supernatant was collected and dried down in MS vials, 50 µl for the amino acids and 200 µl for the polyamines, which were stored at −80 °C until analysis.

#### Amino acid and polyamine derivatization with AccQ-Tag

Extracted samples were derivatized by AccQ-Tag (Waters) according to the manufacturer’s instructions with the following adjustments: the dried extract was dissolved in 20 mM HCl, AccQ•Tag Ultra Borate buffer and the freshly prepared AccQ•Tag derivatization solution 20:60:20 (v/v/v), 100 µl in total for the amino acids and 50 µl in total for the polyamines, and the samples were immediately vortexed for 30 s. Samples were kept at room temperature for 30 min, followed by 10 min at 55 °C. For each batch, quality control samples and procedure blanks were included. Calibration curves were prepared in the same way as the samples.

#### Amino acid and polyamine quantification by LC–electrospray ionization–MS/MS

The derivatized samples were analysed using a 1290 Infinity system (Agilent Technologies), consisting of a G4220A binary pump, a G1316C thermostated column compartment, and a G4226A autosampler with a G1330B autosampler thermostat coupled to an Agilent 6490 triple quadrupole mass spectrometer equipped with a jet stream electrospray source operating in positive ion mode.

Separation was achieved by injecting 1 μl of each sample onto a BEH C_18_ 2.1 × 100 mm, 1.7 μm column (Waters) held at 50 °C in a column oven. The gradient eluents used were H_2_O 0.1% formic acid (A) and acetonitrile 0.1% formic acid (B) with a flow rate of 500 μl min^−1^. The initial conditions consisted of 0% B, and the following gradient was used with linear increments: 0.54–3.50 min (0.1–9.1% B), 3.50–7.0 (9.1–17.0% B), 7.0–8.0 (17.0–19.70% B), 8.0–8.5 (19.7% B), 8.5–9.0 (19.7–21.2% B), 9.0–10.0 (21.2–59.6% B), 10.0–11.0 (59.6–95.0% B), 11.0–11.5 (95.0% B), 11.5–15.0 (0% B). From 13.0 min to 14.8 min, the flow rate was set at 800 μl min^−1^ for a faster equilibration of the column.

The MS parameters were optimized for each compound. Multiple reaction monitoring (MRM) transitions for the derivatized amino acids were optimized using MassHunter MS Optimizer software (Agilent Technologies). The fragmentor voltage was set to 380 V, the cell accelerator voltage to 7 V, and the collision energies ranged from 14 to 45 V; nitrogen was used as the collision gas. Jet-stream gas temperature was 290 °C with a gas flow of 11 l min^−1^, sheath gas temperature of 325 °C and sheath gas flow of 12 l min^−1^. The nebulizer pressure was set to 20 psi, and the capillary voltage was set to 4 kV. The QqQ was operated in dynamic MRM mode with 2-min retention time windows and 500-ms cycle times.

The data were quantified using MassHunter Quantitation software B08.00 (Agilent Technologies), and the amount of each amino acid and polyamine was calculated on the basis of the calibration curves.

### Statistical analysis

Statistical analyses were performed using Microsoft Excel 365 or GraphPad Prism 10. GraphPad Prism 10 or higher was used to generate graphs.

### Accession numbers

Sequence data from this study can be found in the *Arabidopsis* Genome Initiative (www.arabidopsis.org) or GenBank/EMBL databases under the following accession numbers: *AAP5*; AT1G44100.1, *ACT2*; AT3G18780, *BAC1*; AT2G33820.1, *BAC2*; AT1G79900, *ORE1*; AT5G39610. *PIF5*; AT3G59060, *TUB2*; AT5G62690.

### Reporting summary

Further information on research design is available in the Nature Portfolio Reporting Summary linked to this article.

## Supplementary information

Supplementary Information Supplementary Figs. 1–4.

Reporting Summary

Supplementary Table 1 List of primers.

Supplementary Data Source data for Supplementary Figs. 1–4.

## Source data

Source Data Figs. 1–3 All raw data and statistics related to Figs. 1–3.
