# Supplementary material for: The progression of leaf senescence is gated by the cytosolic arginine pool
Source: Nat Plants. 2026 Jun 30;12(7):1325–33. doi: 10.1038/s41477-026-02328-2 (PMC13384897; doi:10.1038/s41477-026-02328-2)
Supplement: Supplementary file 1 — Supplementary Figs. 1–4. [file 41477_2026_2328_MOESM1_ESM.pdf]

nature plants

Supplementary information

https://doi.org/10.1038/s41477-026-02328-2

The progression of leaf senescence is gated by the cytosolic arginine pool

In the format provided by the authors and unedited

Supplementary Figure 1

a

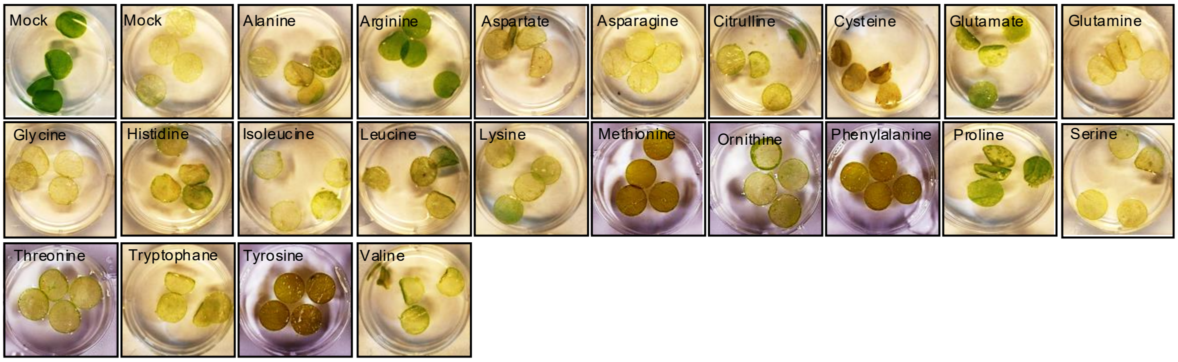

b

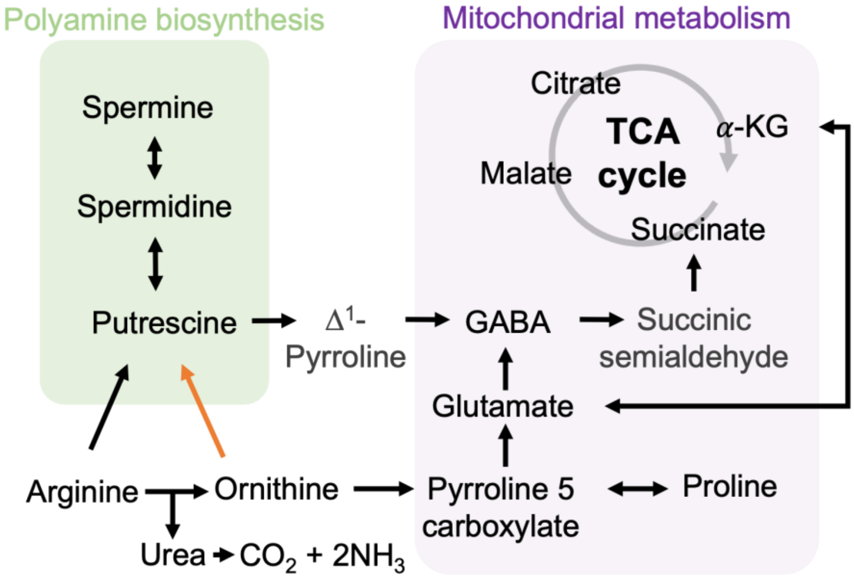

c

Preferred ORE1 Binding Site	Reference	Preferred ORE1 Binding Site in *BAC2* promoter
T[TAG][GA]CGT[GA][TCA][TAG]	Matallana-Ramirez, L. P. et al.[21]	TTACGTGCA

d **e**

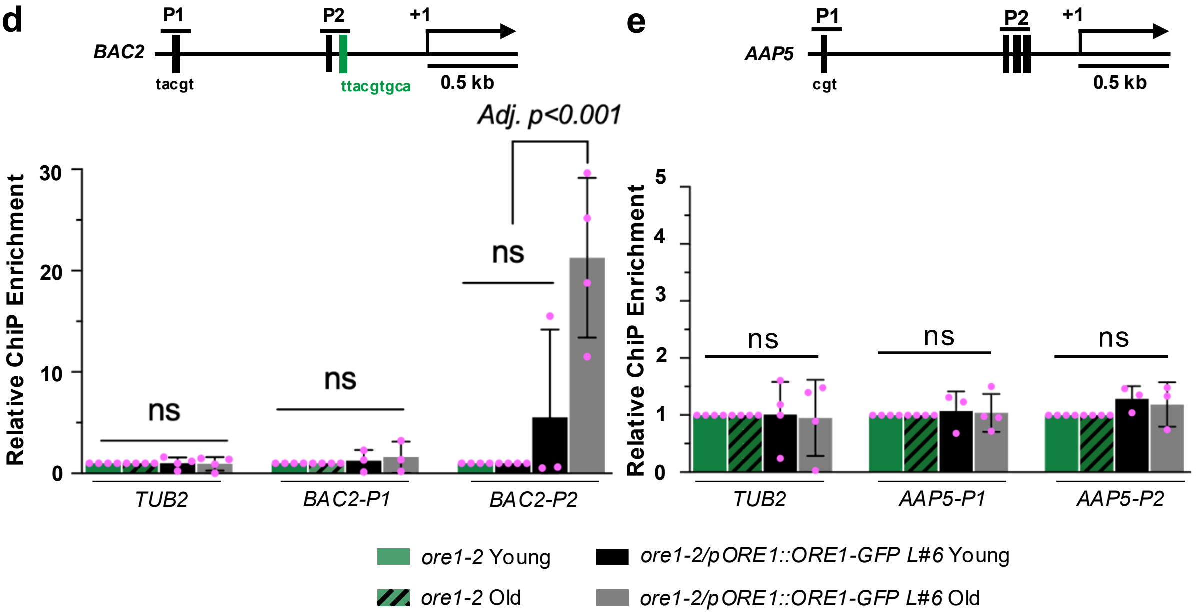

Supplementary Figure 2

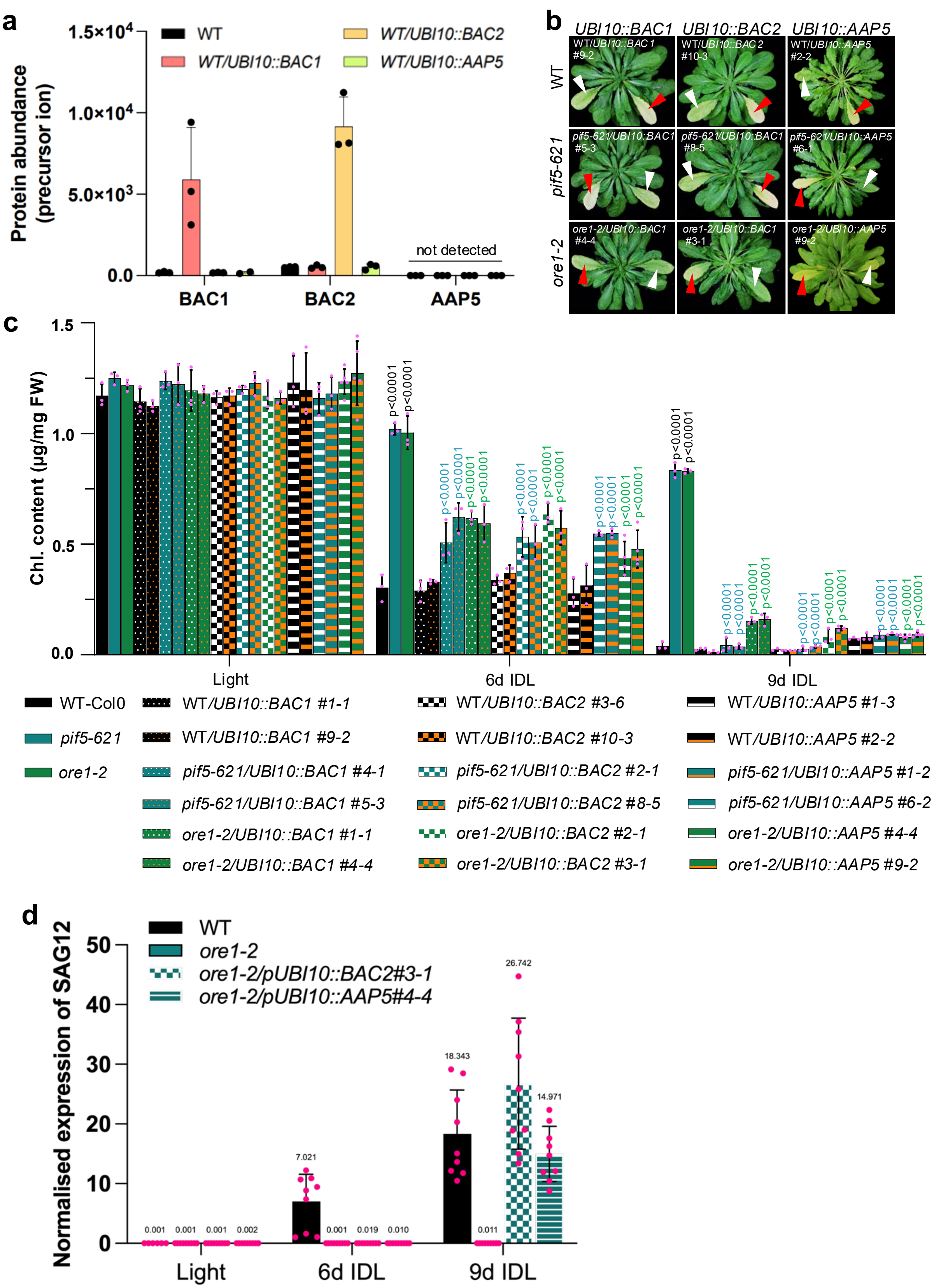

Supplementary Figure 3

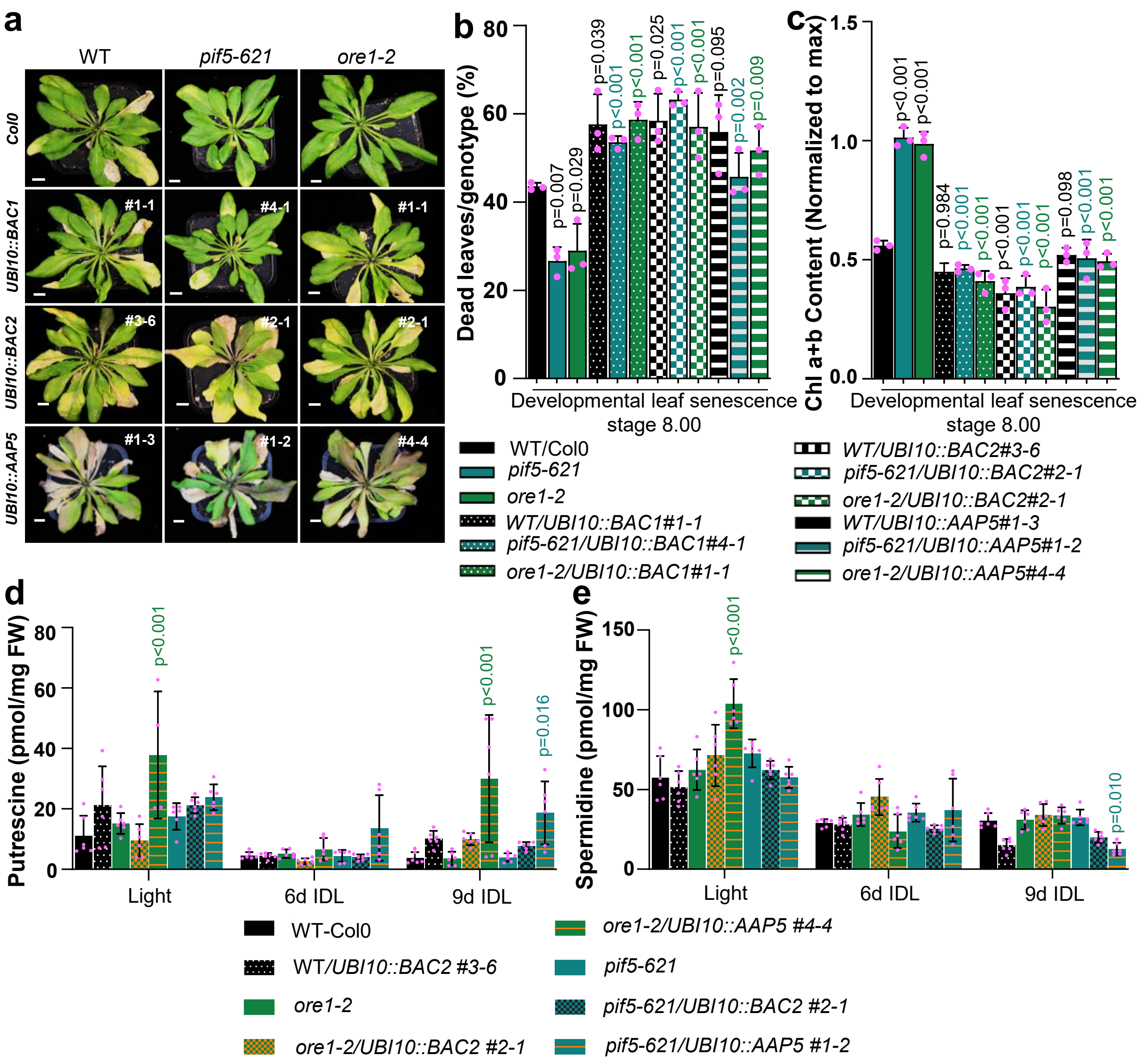

Supplementary Figure 4

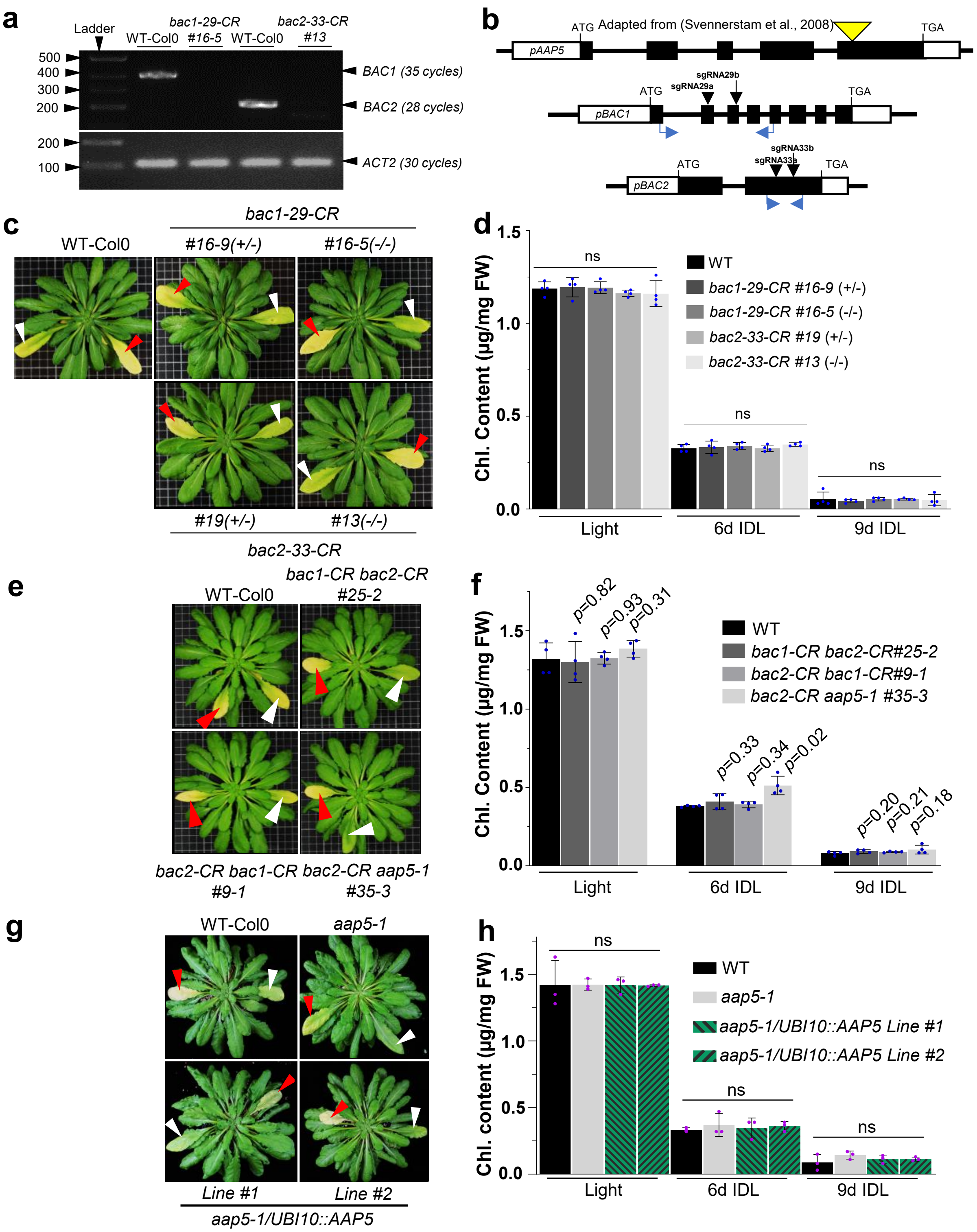

Supplementary figures legends

Supplementary Figure 1. a, Amino acid (AA) feeding experiments on leaf discs. Leaf disc senescence assay of Arabidopsis WT-Col0 plants. Leaf discs were incubated in mock or 1 mM AA solution and kept in the dark for 6 days (*n*=4). **b**, Simplified scheme depicting the metabolic use of arginine in plant cells. Arginine pool supports polyamines (putrescine, spermidine and spermine) and GABA biosynthesis in the cytosol. Moreover, after import to mitochondria, arginine is catabolized to ornithine (with a subsequent release of urea), supporting the biosynthesis of several amino and organic acids. Black arrow: enzymatic reaction, orange arrow: non-enzymatic reaction. Abbreviations: TCA cycle, Tricarboxylic acid cycle; α-KG: alpha-ketoglutarate; GABA: γ-aminobutyric acid; Δ^1-Pyrroline: delta(1)-Pyrroline. **c**, Table with the conserved motif for the ORE1 binding site established by Matallana-Ramirez, L. P. et al.[21], and the sequence of a preferred ORE1 binding site in the *BAC2* promoter (in green). **d**, Chromatin immunoprecipitation (ChIP) assays with an independent line as the one presented in Fig. 1e-f. ORE1 directly binds the promoter of *BAC2* but not of *AAP5*. Schematic diagrams show 1.5 kb upstream sequence of the *BAC2* and *AAP5* promoters, with a translational start sites (ATG) indicated at position +1, respectively. The putative and preferred ORE1 binding sites in the P1 and P2 regions of *BAC2* and *AAP5* promoters were shown in black and green (**d**, **e**, top), respectively. ChIP analyses were conducted using anti-GFP antibodies with chromatin extracted from young (Growth stage#1.12) and old (Growth stage#8.00[22]) *ore1-2* and *ore1-2*/*pORE1*::*ORE1-GFP#6* plants. Subsequently, qPCR analysis was performed using ChIP-DNA as template and primers (Supplementary Table 1) specifically targeting the promoter regions of *BAC2* (**d**; bottom) and *AAP5* (**e**; bottom) genes. *TUBULIN2* (*TUB2*) was used as a control. Data are represented as mean ± S.D., *n* =3 (*ore1-2*/*pORE1*::*ORE1-GFP* Young for P1 and P2, and for Old P1 (BAC2), Old P2 (AAP5)) and *n* =4 for all other samples, independent biological replicates, and individual data points as overlays. Statistical differences were assessed after a two-way ANOVA followed by a Tukey's multiple comparisons test ($p <$ 0.05).

Supplementary Figure 2. a, Protein abundance of BAC1, BAC2 and AAP5* in plants expressing *UBI10*::*BAC1*, *UBI10*::*BAC2* or *UBI10*::*AAP5* in WT backgrounds. Data are represented as mean ± S.D., *n* = 3 independent biological replicates, and individual data points as overlays. *note that the DIA analysis did not allow the detection of AAP5 in any of the samples. **b,** Phenotype of individually-darkened leaves of WT, *pif5-621*, *ore1-2* and complemented lines expressing *UBI10*::*BAC1*, *UBI10*::*BAC2* or *UBI10*::*AAP5* in WT, *pif5-621*

and *ore1-2* background. Leaves were individually darkened for 6 (white arrowhead) or 9 (red arrowhead) days. **c**, Chlorophyll in IDL of plants used in (**b**). Data are represented as mean ± S.D., *n* = 3- 4 independent biological replicates, and individual data points as overlays. Significant differences were determined by two-way ANOVA followed by a Tukey's multiple comparisons test ($p < 0.05$), after comparison with WT (black), with *pif5-621* (teal), or with *ore1-2* (moss green). **d,** Expression of *SAG12* normalised to the expression of *TUB2* in light, and in 6 and 9 days IDL of *WT, ore1-2, and the two complemented lines ore1-2/UBI10*::*BAC2* and *ore1-2/UBI10*::*AAP5*. The above bar values are the mean, *n*= 6 for WT-light (2 independent biological replicates, 3 technical replicates), and *n*= 9 (3 independent biological replicates, 3 technical replicates) for all the other lines.

Supplementary Figure 3. a, Leaf senescence phenotype in response to developmental aging for WT-Col0, *pif5-621* and *ore1-2* and transgenic lines expressing *UBI10::BAC1*, *UBI10::BAC2* and *UBI10::AAP5* in WT-Col0, *pif5-621* and *ore1-2* mutant background. Phenotypes were monitored at growth stage#8.00[1] under long-day conditions. **b**, Age-dependent leaf death quantification of plants used in (**a**). The number of dead leaves in all the selected genotypes was monitored at growth stage#8.00[1]. Leaves were scored as dead when more than half of their blade area had desiccated. The number of dead leaves per genotype (%) is represented as mean values ± S.D. (*n*=3), and individual data points as overlays. **c**, Chlorophyll content from the whole rosette of plants used in (**a**). Data presented here shows mean values, Error bars indicate SD (*n* = 3) and individual data points as overlays. Significant differences were determined by two-way ANOVA followed by a Tukey's multiple comparisons test ($p < 0.05$), after comparison with WT (black), with *pif5-621* (teal) or with *ore1-2* (moss green). **d, e**, Total abundance of Putrescine (**d**) and Spermidine (**e**) in control (Light) and individually-darkened leaves for 6 or 9 days of WT-Col0, WT/*UBI10*::*BAC2, ore1-2, ore1-2*/*UBI10*::*BAC2, ore1-2*/*UBI10*::*AAP5, pif5-621*, *pif5-621*/*UBI10*::*BAC2* and *pif5-621*/*UBI10*::*AAP5*. Data are presented as mean values ± S.D, *n* = 6 independent biological replicates, and individual data points as overlays. Significant differences were determined by two-way ANOVA followed by a Tukey's multiple comparisons test ($p < 0.05$), after comparison with WT (black), with *ore1-2* (moss green), or with *pif5-621* (teal).

Supplementary Figure 4. a, Transcript abundance of *BAC1* and *BAC2* genes in WT-Col0, *bac1-29-CRISPR* (#16-5) and *bac2-33-CRISPR* (#13) lines was determined by semi-quantitative RT-PCR with leaves of 6-week-old rosettes (Supplementary Table

1); *ACTIN2* (*ACT2*) was used as an internal control. **b,** Gene model of *AAP5* with the localisation of the TDNA insertion in the *aap5-1* mutant[15] (top). Gene models of *BAC1* (middle) and *BAC2* (bottom) with the localisation of primers (blue arrows) used in (**a**) for RT-PCR. **c,** Phenotypic characterisation of WT-Col0, *bac1-29-CRISPR* line #16-9(+/-), line #16-5(-/-), and *bac2-33-CRISPR* line#19(+/-), line#13(-/-) after IDL for 6 and 9 days. **d,** Chlorophyll in IDL of plants used in (**c**). **e,** Phenotypic characterisation of WT-Col0, *bac2-CR aap5-1*(#35-3), and *bac1-CR bac2-CR* (#25-2) double mutants after IDL for 6 and 9 days. **f,** Chlorophyll in IDL of plants used in (**e**). **g,** Phenotypic characterisation of WT-Col0, *aap5-1*, and complemented lines expressing *UBI10::AAP5* in *aap5-1* background. **h,** Chlorophyll in IDL of plants used in (**g**). All data are mean values ± SD (*n*=3-4), and individual data points are shown as overlays. Significant differences were determined by *Student's t-test. p* values are indicated on top of each histogram.
